# Embryonic development of pleuropodia of the cicada, Magicicada cassini

**DOI:** 10.1673/2006_06_27.1

**Published:** 2006-10-18

**Authors:** Johannes Strauß, Reinhard Lakes-Harlan

**Affiliations:** Justus-Liebig Universität Gießen, Institute for Animal Physiology, Integrative Sensory Physiology, Wartweg 95, D – 35392 Gießen, Germany

**Keywords:** immunhistochemistry, cell surface molecules, horseradish peroxidase

## Abstract

In many insects the first abdominal segment possesses embryonic appendages called pleuropodia. Here we show the embryogenesis of pleuropodial cells of the periodical cicada, Magicicada cassini (Fisher 1851) (Insecta, Homoptera, Cicadidae). An antibody, anti-horseradish perioxidase (HRP), that is usually neuron-specific strongly marked the pleuropodial anlagen and revealed their ectodermal origin shortly after limb bud formation. Thereafter the cells sank into the epidermis and their apical parts enlarged. A globular part protruded from the body wall. Filamentous structures were marked at the stem region and into the apical dilation. In later embryonic stages the pleuropodia degenerated. Despite the binding of anti-HRP the cells had no morphological neuronal characters and cannot be regarded as neurons. The binding indicates that glycosylated cell surface molecules contribute to the adhesion between the presumably glandular pleuropodial cells. In comparison, anti-HRP does not mark the pleuropodia of Orthoptera.

## Introduction

Cicada embryos possess transiently ventral appendages at the first abdominal segment, the so-called “pleuropodia”. Those pleuropodia have been proposed to be homologous to abdominal legs (Wheeler 1890; Lewis et al. 2000). In accordance with this hypothesis, in many insects a variety of developmental genes involved in patterning the leg anlagen are expressed in both, the thoracic appendages as well as the pleuropodia (Lewis et al. 2000; Yamamoto et al. 2004). However, the pleuropodia are only transient as they degenerate at the end of embryonic development and have no leg-like locomotory function. At least in some insect taxa, the pleuropodia have secretory function (Slifer 1937, 1938; Sander et al. 1985). Ultrastructural studies confirm their glandular structure (Louvet 1973; Rost et al. 2004). It has been proposed that pleuropodial secretions weaken the chorion (Rost et al. 2004) or that they secret a hatching enzyme (Slifer 1938; Engelmann 1957; Louvet 1973). In accordance with the glandular function, indications for the production of the molting enzyme in pleuropodia have accumulated from mechanical evidence in Blattaria (Engelmann 1957) and experimental evidence in Orthoptera (Jones 1956; Novák & Zambre 1974). But the pleuropodia of insects show a large morphological variability and probably diversity in function as well (Sander et al. 1985), even within one taxon as in the Blattaria (Stay 1977; Lambiase et al. 2003), where an osmoregulatory function is most likely (Stay 1977). The pleuropodia of cicadas are anatomically characterized as invaginated (their cell nuclei are located below the epidermis) glandular structures (Wheeler 1889a, b; Heymons 1899). They seem to develop during early organogenesis and differentiate before dorsal closure of the embryo. However, a developmental timing and further functional or histochemical details are unknown.

We analysed immunhistochemically the embryonic development and the formation of the pleuropodia of Magicicada cassini (Fisher 1851) (Insecta, Homoptera, Cicadidae) , a periodical cicada. A developmental series was established that covered the phases of leg formation and dorsal closure. Anti-horseradish perioxidase (HRP), which recognizes glycolysated cell surface molecules on all insects neurons (Jan & Jan 1982; Snow et al. 1987; Haase et al. 2001), was used to follow the development of pleuropodia of M. cassini with respect to the 3-dimensional structure. The immunohistochemical binding by anti-HRP is the first reported for pleuropodia.

## Materials and methods

Immunohistochemical staining was carried out on embryos of M. cassini, which were collected from brood X in summer 2004 in Ohio (United States). Tree branches with freshly laid eggs were cut on June, 26^th^ and stored in plastic containers supplied with tap water to provide humidity. The branches were kept for several weeks at room temperature (22°C). Embryos were dissected from the eggs laid beneath the bark.

As the precise duration of M. cassini embryogenesis is not known (about 52–56 days in the field with unknown temperature regime; Lloyd & Dybas 1966), embryos were dissected consecutively over a period of three weeks in order to cover various stages of embryogenesis, especially from limb bud formation to the end of katatrepsis and the beginning dorsal closure. From these preparations, a developmental series was established that covered the phases of leg formation. Because rearing of embryos could not take place in defined and standardised conditions, the embryos were matched to the developmental stages described for the orthopteran Schistocerca nitens, where these developmental periods correspond to stages from about 30% to 65% of total developmental time (Bentley et al. 1979). The developmental stages of M. cassini were aligned to those of S. nitens by similarities in morphological criteria, mainly position in the egg, relative length, structure of leg anlagen and general morphology, which provided the progress of pleuropodia development without specification of the precise developmental age of the embryos.

For immunohistochemistry, the embryos were fixed in 4% paraformaldehyde in phosphate buffer for one hour. After repeated washing in phosphate buffer they were incubated with the HRP antibody (Cappel Immunocytochemicals, Cochranville, PA) at a concentration of 1: 800 overnight at 4°C. For visualisation of antibody binding, embryos were incubated with a secondary antibody coupled with the chromophore Cy3 (Sigma-Aldrich, www.sigmaaldrich.com) at a concentration of 1:300 for two hours. Control preparations were incubated with only the first or the second antibody. Embryo preparations were then dehydrated in an ethanol series, cleared in methyl salicylate (Merck Inc., www.merck.com) and embedded in Entellan medium (Merck). The preparations were examined using a Leitz Dialux 20 fluorescence microscope. Photographs were taken using a digital camera system (Intas GmbH, www.intas.de/; 768x1024 pixel) and digitally revised in Adobe Photoshop, by enhancing contrast and inserting scale bars. Some preparations were optically sectioned with a confocal microscope (Leica, www.leica-microsystems.com, TCS SP2).

## Results

**Figure 1:**
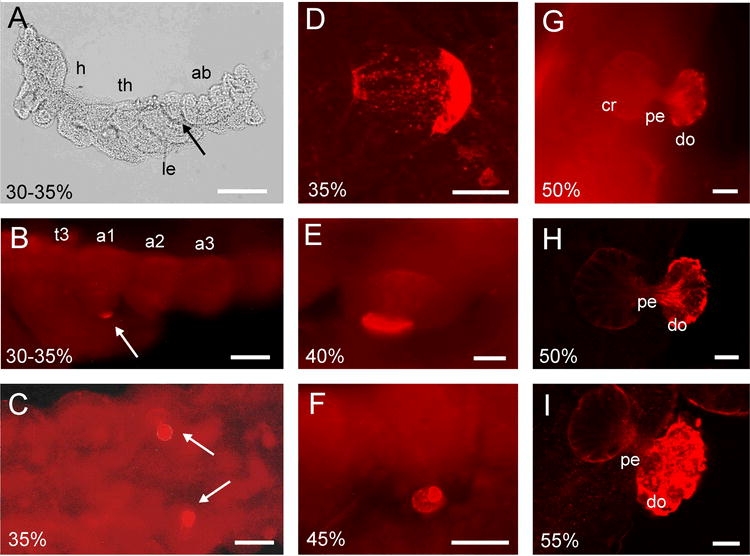
Embryonic developmental of the pleuropodium of Magicicada cassini as revealed by anti-HRP immunhistochemistry. The embryonic development has been staged in percent according to that of Schistocerca nitens (Bentley et al. 1972). **A**; Brightfield micrograph of an embryo of 30–35% of embryonic development. ab: abdomen (the distal segments are missing), h: head, le: leg anlagen, th: thorax. **B to I**; Anti-HRP immunhistochemistry of the different developmental stages of pleuropodia. a1-a3: abdominal segments 1 – 3, cr: crescent, do: dome, pe: pedunculus, t3: metathoracic segment. The arrows point to the pleuropodia in the first abdominal segment. Scales: A: 100μm, B,C,F: 50μm, D,E,G,H,I: 20μm.

The first documented embryonic stage of M. cassini corresponds to 30–35% of embryonic development of S. nitens (Fig. 1A; Bentley et al. 1979). The embryo is dorsally open and short limb buds are present without a clearly visible segmentation. During this stage a pleuropodium is not visible from the outside, but the immunostaining revealed a first anti-HRP signal at the ventro-lateral margin of the first abdominal segment (Fig. 1B). This situation represents the earliest antibody binding to pleuropodia and is likely to correspond to a very early stage of pleuropodium development. The immunostaining is bilateral symmetrical and restricted to the first abdominal segment (Fig. 1C). Only the apical surfaces of six to eight cells were immunreactive. Shortly thereafter anti-HRP binding extended throughout the whole epidermis, still labelling the apical part of the about cells most strongly (Fig. 1D). At about 40% of embryonic development, the cells elongate and sink into the body cavity (Fig. 1E). Their nuclei are located at the basal part within the body cavity. By about 45% of development the apical parts evaginate above the epidermis (Figs. 1F,G). Consequently, the pleuropodium is morphologically separated into a basal crescent, a narrow pedunculus extending through the epidermis and an external dome. This separation becomes even more obvious at around 50% of development. This stage is characterized by an extension of the hind legs to the anterior margin of the third abdominal segment and the beginning of the dorsal closure. The dorsal view shows that anti-HRP binding is still strongest in the apical, globular dome that protrudes from the body wall (Figs. 1G-I). Confocal microscopy demonstrates the strong polarity of the pleuropodial cells and the restriction of anti-HRP binding to the cell surfaces (Fig. 1H). Beneath the globular dome lies a constricted domain with filamentous structures (the peduncuclus), which links to a crescent inside of the body wall. These filamentous structures showed a relatively strong labelling, perhaps due the dense clustering of cell membranes (Fig. 1H). The interior crescent shows about 40 cell bodies with the blank space of the nuclei beneath the epidermis. The cell surfaces extruding from the epithelium enlarge and display a inhomogeneous, granular structure with a strong anti-HRP binding (Fig. 1I). Shortly thereafter the pleuropodia degenerate, although it could not be followed in detail by immunhistochemical marking. The subsequent secretion of cuticle prevents the penetration of the antibodies.

Interestingly, the marking of neuronal structures in the embryos was rather weak, although present. In most preparations of embryos between 35% and 50% a staining of some peripheral axons and neuronal cell bodies could be detected (data not shown).

## Discussion

This study contributes to the development of cicadas, which is only rarely investigated. After the landmark studies in Hemiptera (Wheeler 1889a, b; Heyman 1899) including the periodical cicada, Cicada septemdecim, now recognised as three species (Alexander & Moore 1962), only circumstantial evidence concerning embryogenesis of cicadas has been reported. Embryonic development is estimated from cross-species breeding experiments to take about 52–56 days (Lloyd & Dybas 1966). In this report embryonic development in M. cassini was related to criteria used for the grasshopper S. nitens (Bentley et al. 1979), which seems reasonable due to many morphogenetic similarities in embryogenesis. Development of the pleuropodium was followed using an anti-HRP immunochemical marker that was previously not known to bind to abdominal appendages.

### Insect pleuropodia

Adult insects are characterized by the absence of legs on the abdomen. By contrast embryos and juvenile stages often possess various appendages on the abdomen, including pleuropodia. The function and ontogenetic origin of insect pleudopodia has not been well characterized. Pleuropodia express, at least transiently, many genes involved in limb formation, such as Distalless (dll), dachshund and SP8 (Lewis et al. 2000; Beermann et al. 2004; Yamamoto et al. 2004). The development of a limb seems to be suppressed by the homeotic gene Hox gene Abd-A (Lewis et al. 2000). Pleuropodia are similar to the legs in their ectodermal origin (Wheeler 1889a, b; our results). Some insects have evaginated pleuropodia (Louvet 1976) whereas many species have invaginated pleuropodia (Wheeler 1889a, b; Rost 2004). Our results show the ectodermal origin and the invagination process for M. cassini. Immunpositive cells are first located within the epidermis. Thereafter the number of cells increases and the cells sink into the body cavity. During this process morphogenesis of pleuropodia in M. cassini is further signified by polarisation between the apical and basal parts, and the appearance of the pedunculus. A migration of cells and their nuclei has also been seen in Blattaria (Lambiase et al. 2003) but further studies are needed for a comparative analysis. The morphogenesis of pleuropodia takes place mainly between 30 – 50% of embryonic development (see Bentley et al. 1979 for reference to developmental time), which relates to the onset of katatrepsis until dorsal closure. These data are consistent with the developmental stages reported previously (Wheeler 1890).

### Function

Pleuropodia display a large diversity in morphology (Sander et al. 1985; Lambiase et al. 2003). Correspondingly, evidence and speculation about their function varies and the functional relevance of pleuropodia is not clearly established in any insect. In insects, including M. cassini, the pleuropodia degenerate during embryogenesis restricting their function to the embryo. Different functions have been proposed and discussed (Wheeler 1890). Pleuropodia have been proposed to have glandular function by secreting a hatching enzyme (Engelmann 1957; Novák & Zambre 1974; Sander et al. 1985). Based on ultrastructural and morphological evidence an osmoregulatory function has been proposed for the strikingly long pleuropodia of Blattaria (Stay 1977). New techniques in molecular biology will hopefully reveal unequivocal evidence of function, which might be different in different species. It remains to be shown whether the formation of a gland is controlled by genes like *dll* or whether its expression in pleuropodia is an evolutionary rudiment. A first indication could be a coexpression of *dll* and the HRP antigen, which has not yet been done due to the sporadic availability of embryos of a 17-year periodical cicada.

### Anti-HRP-immunoreactivity

The antibody used in this investigation against horseradish peroxidase binds specifically to cell surface glycoproteins on neurons in the developing and adult nervous system of insects (Jan & Jan 1982; Snow et al. 1987). A neuronal anti-HRP binding has been proposed for all ecdysozoans (Haase et al. 2001). The cicada M. cassini is no exception, although the neuronal marking is rather weak, compared to that of the pleuropodium. Generally, anti-HRP-antibody immunohistochemistry is not restricted to neurons and glia cells but in rare cases includes glandular structures, such as the garland cells of Drosophila (Jan & Jan 1982). The pleuropodial marking reported here is another case of anti-HRP binding to a secretory cell type. Interestingly, in other insects, such as the grasshoppers Schistocerca gregaria, Chorthippus biguttulus or the bushcricket Mecopoda elongata, no pleuropodial marking with anti-HRP was detected (Snow et al. 1987; Lakes-Harlan & Pollack 1993; unpublished results). This could indicate an anatomical or functional modification in cicadas.The immunhistochemical signal does not represent a neuronal marking in the pleuropodium of M. cassini. Neither axonal nor dendritic structures have been detected in any developmental stage within the pleuropodium.

The cellular function of anti-HRP epitopes in cicada pleuropodia remains poorly understood but may be speculated upon. Anti-HRP binds to cell adhesion molecules including the ontogenetically important Fasciclin I and II (Bastiani et al. 1987). Cell adhesion molecules attach cells or cell domains like axons to each other. For pleuropodia, the function of cell surface molecules detected by anti-HRP may be the compaction of the secretory cells. Ultrastructure studies have demonstrated that the cells are, at least in early stages, tightly attached to each other by adherens junctions in the apical domain (Louvet 1973, 1976,1977). A significant immunoreactivity of anti-HRP marking occurs in the filamentous structures of the pleuropodium. In this structure adhesive cell surface molecules may be an additional mechanism to the described adherens junctions by creating a high density of cellular processes. Apically, the constricted pedunculus broadens into the dome. The strong marking at the dome might be due to glycolysated carbohydrates associated with the proposed secretory function.
